# The Role of Selenium in Health and Disease: Emerging and Recurring Trends

**DOI:** 10.3390/nu12041049

**Published:** 2020-04-10

**Authors:** Catherine Méplan, David J. Hughes

**Affiliations:** 1 School of Biomedical, Nutritional and Sport Sciences, Newcastle University, Newcastle upon Tyne NE1 7RU, UK; 2 Cancer Biology and Therapeutics Group, UCD Conway Institute, School of Biomolecular and Biomedical Science, University College Dublin, D04 V1W8 Dublin, Ireland; david.hughes@ucd.ie

In this Special Issue of *Nutrients*, “The Role of Selenium in Health and Disease” covers diverse diseases in the 8 original research articles and 2 reviews, such as cardiovascular disorders (CVD), metabolic syndrome, obesity, cancer, and viral infection, and highlights novel potential biomarkers of disease risk and prognosis. From a public health perspective, the different manuscripts emphasize the already known U-shaped dose-response relationship between selenium (Se) concentration and disease risk across different study populations from Europe [1,2,3], the Middle East and North Africa [4], and Taiwan [5]. These reports therefore strengthen the importance of developing more personalized nutritional advice targeted at individuals at risk of disease with low Se intake. The results presented in this *Issue* also emphasize a need for further research into the mechanisms by which Se levels, selenoprotein expression, and inherited genetic variation in selenoproteins and interacting pathway genes may affect molecular pathways that either prevent or contribute to disease development. This is explored in human cohort studies [1,2,3,5,6] and in animal [7] and in vitro models [8]. 

## 1. Selenium, Cardiovascular Diseases, Metabolic Syndrome, and Obesity

Although links between Se levels, selenoprotein expression, and CVD or lipid metabolism are well established [1,2], the mechanisms supporting these associations remain unclear. Two of the manuscripts presented in this *Issue* established, in distinct Northern European populations, two novel Se-related biomarkers of risk and prognosis for CVD. Schomburg et al. identified, in a Swedish human perspective cohort, a strong correlation between low baseline plasma levels of Selenoprotein P (SELENOP; the major Se-transporter) and risk for all-cause mortality, CVD mortality, and a first CVD event during the follow-up period of 9.3 (8.3–11) years [2]. Based on the known high prevalence (20%) of low SELENOP levels in the Northern European population [2,3], these data suggest that plasma SELENOP concentrations may be used as an early biomarker of CVD risk and, therefore, that targeted Se supplementation could be used as a preventative measure. However, further studies are required to determine the causal link between SELENOP levels and cardiovascular events and to assess whether genetic variants in the *SELENOP* gene, known to influence Se bioavailability, affects this correlation.

Non-selenoprotein Se-binding proteins, such as Selenium-Binding Protein 1 (SELENBP1), have been underappreciated in Se-related research, considering their significant relevance for major physiological processes and that SELENBP1 has been previously implicated in myocardial infarction and poor clinical outcomes from various tumour types [1]. In a distinct cohort of 75 German patients undergoing elective cardiac surgery, a marked but transient increase in circulating SELENBP1 concentrations during the surgical process was correlated, in most patients, with the duration of ischemia and myocardial damage. This suggests that serum concentrations of SELENBP1 could constitute a quantitative marker for myocardial hypoxia [1]. On the contrary, high serum Se levels were associated with metabolic syndrome and insulin resistance markers in a large Taiwanese cohort, although markers of adiposity and lipid functions varied by sex [5]. 

Further evidence of the importance of Se in lipid and energy metabolism is supported by the observation that the enzyme selenocysteine lyase (Scly) is involved in weight gain, resistance to anorexigenic hormone leptin, and thermogenesis [7]. Scly is responsible for the breakdown of intracellular selenocysteine to alanine and selenide, which is in turn recycled for the synthesis of new selenoproteins. The conditional Scly knockout (KO) model affecting agouti-related peptide-positive neurons in the hypothalamus (Scly-Agrp KO mice) displayed a reduction in high fat diet-induced weight gain and protection against development of leptin resistance compared with controls (mice expressing Scly). This was linked to progressive degeneration of some Agrp neurons and in brown adipose tissue pattern [7].

Taken together, these data provide some novel potential targets for understanding the mechanisms by which Se affects CVD or lipid metabolism and for further investigating the potential of SELENOP and SELENBP1 as CVD biomarkers.

## 2. Selenium and Cancer

Accumulating experimental and observational evidence suggests that insufficient Se intake and/or selenoprotein genetic variations may contribute to the development of several tumours including colorectal cancer (CRC), mediated by oxidative and inflammatory stress response selenoproteins [3].

Higher Se status levels (total serum Se levels and SELENOP concentrations) were previously reported in a multi-centre, European prospective cohort study (EPIC) to be associated with a decreased CRC risk. The manuscript in this issue by Fedirko et al. extends this work by describing the largest association with CRC risk for common genetic variations related to Se metabolism in approximately 1400 cases and 1400 controls within this EPIC cohort. The study examined over 1000 single nucleotide polymorphisms (SNPs) in 154 genes within the Se biological pathway (including all selenoprotein genes and Se metabolic pathway genes) and interactions with serum Se status biomarkers from the previous study. The findings provide the most comprehensive evidence to date that individual genotypes relevant for selenoprotein expression, metabolism, and function and interaction with Se status may affect CRC risk in a population of marginally low Se status, such as in Europe. Pathway analyses indicated that, for genes in antioxidant/redox and apoptotic pathways, the influence of SNPs on the disease risk is also dependent on interaction with Se status [3].

Addressing the sparse data on selenoprotein expression in CRC, Hughes and colleagues assessed selenoprotein gene transcript levels in the neoplastic and matched mucosal tissue from Irish and Czech colorectal adenoma (CRA) and CRC patients and examined the interaction with Se status levels [6]. Several selenoproteins (including biological stress response and Se biosynthesis genes) were differentially expressed in the disease tissue compared to the normal tissue of both CRA and CRC patients, and that also showed tumour gene expression changes correlated to levels of Se or SELENOP. Across the disease tissues from the adenoma and both cancer groups, *GPX2* and *TXNRD3* exhibited higher expression while *GPX3*, *SELENOP*, *SELENOS*, and *SEPHS2* showed lower expression. The authors concluded that selenoprotein expression changes could be used as biomarkers of functional Se status and the colorectal adenoma to cancer transition. In survival analyses, only a higher *SELENOF* expression was associated with poorer survival outcomes after cancer diagnosis. Although this did not retain significance after multiple testing correction, there is possible biological validity to this observation as *SELENOF* has been previously linked with oncogenesis [6].

Moving to innovative Se metabolism cell-line experiments, Sonet et al. suggest that selenized lipids from plant oils (selenitriglycerides; Selol), proposed to have antineoplastic effects, may provide natural Se supplementation as a bioavailable selenocompound with lower toxicity than chemical forms like selenite [8]. The authors showed that Selol could be an efficient source of Se for selenoprotein biosynthesis in immortalized kidney (HEK293) and prostate cancer (LNCaP) cell lines but not in immortalized prostate cells (PNT1A,) possibly due to variance in lipid metabolism between the different cell lines [8]. As transformation of various chemical species into selenide is the gateway step for further incorporation into selenoproteins, cell-specific Se metabolism to selenide via selenized triglycerides requires further study.

## 3. Selenium and Responses to Stress

The importance of Se status in modifying the ability of an individual to respond to stress has been linked to the development of many diseases including CVD [1,2], cancers [3,6], and neurodegeneration [9]. As a number of selenoproteins play a crucial prevalent role in the response to oxidative stress and the control of redox status [3,10] as well as in the maintenance of endoplasmic reticulum (ER) stress response [9], mechanisms that alter the expression of these selenoproteins have the potential to lead to an increased risk of disease.

In a currently highly pertinent review, considering the current pandemic of the SARS-CoV-2 virus mediated COVID-19 disease as we write, Guillin et al. discuss the role of Se in protection from viral contagion [10]. During viral infection, the pathogens induce oxidative stress by generating reactive oxygen species and by altering the cellular antioxidant defences, including selenoproteins such as glutathione peroxidases (GPx) and thioredoxin reductases. Consequently, the host’s Se requirements increase and, in hosts deficient in Se, the oxidative stress can induce viral genome mutations, leading to increased microbial virulence (e.g., coxsackie and influenza viruses). Other mechanisms by which the host’s nutritional status can affect viral infection progression include reducing the ability of the immune system to respond to the virus (e.g., human immunodeficiency virus (HIV) and hepatitis C and B viruses). In silico data have also revealed the presence of selenoprotein genes in the genomes of several common viruses (e.g., HIV) that resemble mammalian GPx. The function and regulation of such viral selenoproteins remains unclear but could afford viruses protection from oxidative damage [10].

Due to the crucial role of ER stress in many cellular processes, understanding the consequences of alteration of components of the ER stress response has the potential to lead to the discovery of new therapeutic/nutritional targets. Seven selenoproteins are known to be present in the ER, but not all have been well characterised. In a timely review, Ren et al. discuss the current knowledge on ER-resident SELENOF, including its function and role in ER stress response and the regulation of its expression [9]. The review also summarises results from genetic association studies linking genotypes for SNPs in the *SELENOF* gene to risk for various cancers, Kashin–Beck disease, and AIDs progression, with a particular focus on two well-characterised functional SNPs (rs5845 and rs5859) affecting SELENOF protein expression. Furthermore, the authors discuss the dysregulation of SELENOF expression in several tissue and pathologies, from cancer to neurodegeneration and immune system diseases [9]. Future studies investigating the role of other ER-resident selenoproteins could lead to a better understanding of mechanisms that contribute to the development of a wide range of common complex diseases.

Overall, the studies in this Special Issue strengthen and broaden the evidence base that the risk of several chronic diseases and viral infections may be modified by Se status, genotype, sex, and gene variation interactions within biological pathways. Detailed investigation of Se intake levels and metabolism is needed to more fully elucidate the relevance for disease etiopathogenesis, especially for populations with diverse Se status levels and/or individuals with potentially at-risk disease or protective Se pathway genotypes.
